# Alveolar recruitment maneuver attenuates extravascular lung water in acute respiratory distress syndrome

**DOI:** 10.1097/MD.0000000000007627

**Published:** 2017-07-28

**Authors:** Fu-Tsai Chung, Chung-Shu Lee, Shu-Min Lin, Chih-Hsi Kuo, Tsai-Yu Wang, Yueh-Fu Fang, Meng-Heng Hsieh, Hao-Cheng Chen, Horng-Chyuan Lin

**Affiliations:** a Department of Thoracic Medicine, Saint Paul's Hospital, Taoyuan; b Department of Thoracic Medicine, Chang Gung Memorial Hospital at Linkou, Chang Gung University, College of Medicine, Taipei; c Graduate Institute of Clinical Medical Sciences, College of Medicine, Chang Gung University, Taoyuan; d Division of Pulmonary Medicine, Department of Internal Medicine, Shuang Ho Hospital, Taipei Medical University, New Taipei City, Taiwan.

**Keywords:** acute respiratory distress syndrome, and outcomes, extravascular lung water, oxygenation, recruitment maneuver

## Abstract

**Background:**

The alveolar recruitment maneuver (RM) has been reported to improve oxygenation in acute respiratory distress syndrome (ARDS) and may be related to reduced extravascular lung water (EVLW) in animals. This study was designed to investigate the effects of RM on EVLW in patients with ARDS.

**Methods:**

An open label, prospective, randomized controlled trial including patients with ARDS was conducted in hospitals in North Taiwan between 2010 and 2016. The patients were divided into 2 groups (with and without RM). The primary endpoint was the comparison of the EVLW index between the 2 groups.

**Results:**

Twenty-four patients with ARDS on mechanical ventilator support were randomized to receive ventilator treatment with RM (RM group, n = 12) or without RM (non-RM group, n = 12). Baseline demographic characteristics were similar between the 2 groups. After recruitment, the day 3 extravascular lung water index (EVLWI) (25.3 ± 9.3 vs 15.5 ± 7.3 mL/kg, *P* = .008) and the arterial oxygen tension/fractional inspired oxygen ratio (PaO_2_/FiO_2_) (132.3 ± 43.5 vs 185.6 ± 38.8 mL/kg, *P* = .003) both improved over that of day 1. However, both EVLWI and PaO_2_/FiO_2_ did not significantly change from day 1 to 3 in the non-RM group.

**Conclusion:**

RM is a feasible method for improving oxygenation and the EVLW index in patients with ARDS, as well as for decreasing ventilator days and intensive care unit stay duration.

**Key Points**

- The recruitment maneuver is a feasible treatment for acute respiratory distress syndrome (ARDS).
- The recruitment maneuver could improve the extravascular lung water index (EVLWI) of patients with ARDS.
- The recruitment maneuver is a feasible method for improving oxygenation and EVLWI in patients with ARDS, as well as decreasing ventilator use and the length of ICU stay.
- The biomarkers and treatment strategies of improvement in ARDS may be developed from the results of this study. EVLWI-lowering therapy with RM may improve oxygenation and vascular permeability in ARDS. Future research is needed to discover the possible underlying mechanisms of ARDS and vascular permeability and develop a novel treatment.

## 1 Introduction

The main features of acute respiratory distress syndrome (ARDS) are loss of lung volume^[1]^ and the accumulation of alveolar and interstitial fluid in the lungs, which causes severe lung edema reflected as elevated extravascular lung water (EVLW).^[2]^ An EVLW-induced mismatch of the ventilation-perfusion ratio results in severe hypoxemia and is associated with a high mortality rate in patients with ARDS.^[3]^

Lung-protective ventilation with a low tidal volume and positive end-expiratory pressure (PEEP) can avoid lung over-distension and the opening and closing of small airways and alveoli in ARDS.^[4,5]^ In particular, a low tidal volume applied in ARDS has been shown to effectively improve oxygenation, reduce lung injury, and increase survival among these patients^[6]^; however, it may also cause some alveolar collapse and even refractory hypoxemia. In ARDS, accumulation of EVLW may result in lung tissue disruption, thus requiring higher PEEP to counteract the gravity-related lung collapse and consolidation.^[7]^

Accordingly, the ventilator approach to ARDS has always included the application of PEEP to avoid lung collapse.^[8,9]^ The alveolar recruitment maneuver (RM) has been shown to be an essential clinical treatment,^[10,11]^ as lung atelectasis, a hallmark of ARDS. Alveolar RM refers to the dynamic process of reopening unstable, airless alveoli through an intentional transient increase in transpulmonary pressure. RM reopens collapsed lung tissue via an intermittent, short-acting increase in airway pressure, which may improve oxygenation in patients with ARDS.^[12–14]^ Animal studies have also shown that alveolar RM can improve oxygenation and reduce the EVLW index (EVLWI).^[15]^

This study was designed to investigate the effects of RM on respiratory mechanics and EVLWI in patients with ARDS. By investigating the relationship between EVLWI and RM in patients with ARDS, future studies may develop new therapies to restore the fine biological balance between epithelial and endothelial mechanisms that are disrupted during the life-threatening processes that lead to ARDS-related mortality.

## 2 Methods

### 2.1 Ethics

The Institutional Review Boards of the study hospitals approved this study (IRB No.: 99-0362A3) and written informed consent was obtained from all patients. The study has been registered on the ClinicalTrials.gov website (NCT01552070).

### 2.2 Patients

From November 2010 to January 2016, patients who met the standard published criteria for ARDS were enrolled in the study and recruited consecutively. Patients were randomized (1:1) to either receive or not receive the RM. The procedures of random allocation, enrolling participants, and assigning participants to interventions were conducted by the principal investigators. Patients were followed up until death or discharge from the hospital. All patients receiving endotracheal mechanical ventilation for hypoxemic acute respiratory failure were eligible if the following criteria were met for no more than 48 hours before enrollment: arterial oxygen tension [PaO2]/fractional inspired oxygen [FiO2] ratio (PaO2/FiO2) <200 mm Hg at time of enrollment, recent appearance of bilateral pulmonary infiltrates consistent with edema, and no clinical evidence of left atrial hypertension (pulmonary-capillary wedge pressure more than 18 mm Hg, when available). Exclusion criteria were age <20 years, known pregnancy, participation in another trial within 30 days before meeting the eligibility criteria, severe chronic respiratory disease requiring long-term oxygen therapy or home mechanical ventilation, pneumothorax, expected duration of mechanical ventilation <48 hours, and a decision to withhold life-sustaining treatment.

### 2.3 Baseline assessments

All eligible patients were enrolled within 48 hours of meeting ARDS criteria. Patient-specific data were obtained upon enrollment, including demographic data, past medical history, and Acute Physiology and Chronic Health Evaluation II (APACHE II) score. Thereafter, time points are defined relative to the day of randomization. In addition, relevant medical history was collected, including chronic airway diseases (asthma, chronic airway obstructive disease, and bronchiectasis), cardiovascular disease (hypertension, cerebral stroke, coronary disease, heart failure, and arrhythmia), and chronic renal failure. Physiological parameters, including the previous 24-hour net fluid balance (input/output) and shock status, were assessed. Shock was defined as a systolic blood pressure <90 mm Hg or mean arterial pressure <60 mm Hg and requiring vasopressor use. Patient management decisions, including the type and amount of volume resuscitation, were based on the discretion of the primary intensive care physician. Laboratory serological data (albumin, white blood cell counts, and platelet counts) and oxygenation parameters (PaO_2_/FiO_2_, lung injury score, and chest radiograph score) were given simultaneously as EVLWI was made available by the pulse contour continuous cardiac output (PICCO) system. Ventilator parameters collected were tidal volume indexed to predicted body weight (PBW), PEEP, lung compliance, plateau pressure, mean airway pressure, and minute volume on day 1.

### 2.4 Ventilator settings

#### 2.4.1 (1) General setting

The general ventilator setting was used by the pressure-controlled mode and the PEEP was titrated by the level of FiO_2_ to keep the saturation of oxygen (SaO_2_) more than 90%. The level of pressure control was adjusted to maintain low tidal volume (6–8 mL/kg predicted body weight [PBW]) strategy. The respiratory rate and tidal volume were setting to avoid unstable hemodynamic data and adjusted by blood gas data and lung mechanics.

#### 2.4.2 (2) Recruitment maneuver

Under pressure-controlled mode, driving pressure was set to 15 cm H_2_O above PEEP. When the recruitment phase was started, the level of PEEP was increased to a maximum of 40 cm H_2_O in increments of 5 cm H_2_O from 10 cm H_2_O, and the level was fixed to 40 seconds in each increment. Then, the ventilator was transferred to PEEP-titration phase, the PEEP was adjusted to 25 cm H_2_O, and it was reduced by increments of 5 cm H_2_O each time. The each increment was fixed to 5 minutes till the end-maneuver PEEP. During the phase of recruitment, the titration of PEEP was interrupted when the recruited lung was reduced to the percentage more than 2% from the attained maximal recruitment. This was pretending as a constant pulmonary inflation maneuver, with a positive ventilator pressure of 40 cm H_2_O applied for 40 seconds. The end-maneuver PEEP was set to 10 cm H_2_O. The RM was performed only once on day 1 when the patients enrolled. After RM, the ventilator setting was adjusted to general setting before RM. Hemodynamic data, gas exchange, and lung mechanics were measured at the end of RM and again 15 minutes later with EVLWI. All patients received a chest radiograph to identify extra-alveolar air within 24 hours after RM. Despite someone concerned the higher airway pressures,^[16]^ a report also revealed that the implementation of higher PEEP strategies with constant driving pressure may not cause adverse outcomes.^[17]^

### 2.5 Measurement of extravascular lung water

The measurement of EVLW reported in previous studies.^[18,19]^ Briefly, it was based on the transpulmonary thermodilution method. We used only one single indicator (with cold saline solution) and established a satisfactory correlation with the gravimetric method.^[20]^ A commercialized catheter (PulsiocathPV2014L16; Pulsion Medical Systems, Munich, Germany) was placed in the descending aorta via the femoral and a standard central venous catheter were connected to pressure transducers and an integrated bedside monitor (PICCO; Pulsion Medical Systems). With 3 successive central venous injections of 10 mL iced 0.9% saline solution, both continuous cardiac output (CO) calibration and EVLW level were achieved.

### 2.6 The primary and secondary outcome measures

The primary outcome measures were EVLWI on days 1 to 7. The secondary outcome measures were oxygenation parameters (PaO_2_/FiO_2_, lung injury score, and chest radiograph score), ventilator days, length of stay in the ICU (days), ICU mortality, and in-hospital mortality after admission to the ICU. No changes were made to the trial outcomes.

### 2.7 Statistical analysis

We planned to enroll 12 patients in each group, which would provide a power of 80% to detect the difference in EVLWI between RM and non-RM groups, using a 5% level of significance. This potential effect on EVLWI was estimated based on the potential mechanisms of RM effects as determined in a preclinical study ^[19]^.

All data were expressed as the mean ± standard deviation or 95% confidence interval (CI) and number (%). Since the sample size was small, non-parametric tests were used in the study. Quantitative variables between 2 groups were then compared using the *t* test for continuous and ordinal variables and the chi-square test for nominal variables. Data among different time points were compared using a 2-way analysis of variance (ANOVA). A 2-sided

*P-*value of <.05 was considered statistically significant. All analyses were conducted using the Statistical Package for the Social Sciences (SPSS) software (version 17.0, SPSS, Chicago, IL) and Prism 5 for Windows (version 5.03, Graphpad Software, Inc., San Diego, CA).

## 3 Results

**Figure 1:**
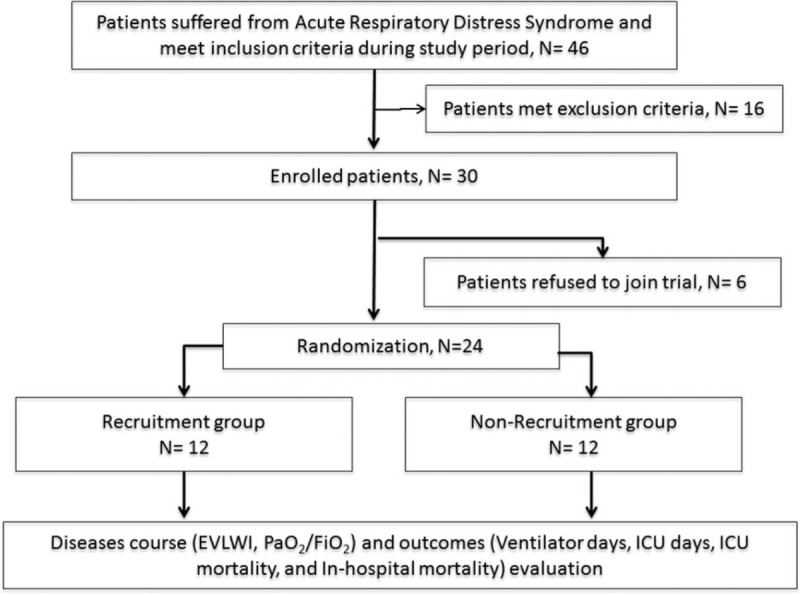
Overview of the included patients.

Duringthe study period, 49 patients with ARDS were evaluated, 18 met the exclusion criteria and were excluded, and 7 of the remaining 31 patients refused to participate; thus, a total of 24 patients were enrolled for randomization (Fig. 1). In this cohort, all patients required mechanical ventilation. Table 1 lists the baseline characteristics of the patients with and without RM on day 1. Variables including age, gender, APACHE II score, body mass index (BMI), the fluid balance from the previous 24 hours, vasopressor use, EVLWI, PaO_2_/FiO_2_, lung injury score, PEEP, lung compliance, plateau airway pressure, mean airway pressure, tidal volume, and minute ventilation are shown. The baseline demographics of the 2 groups were not significantly different.

**Table 1:**
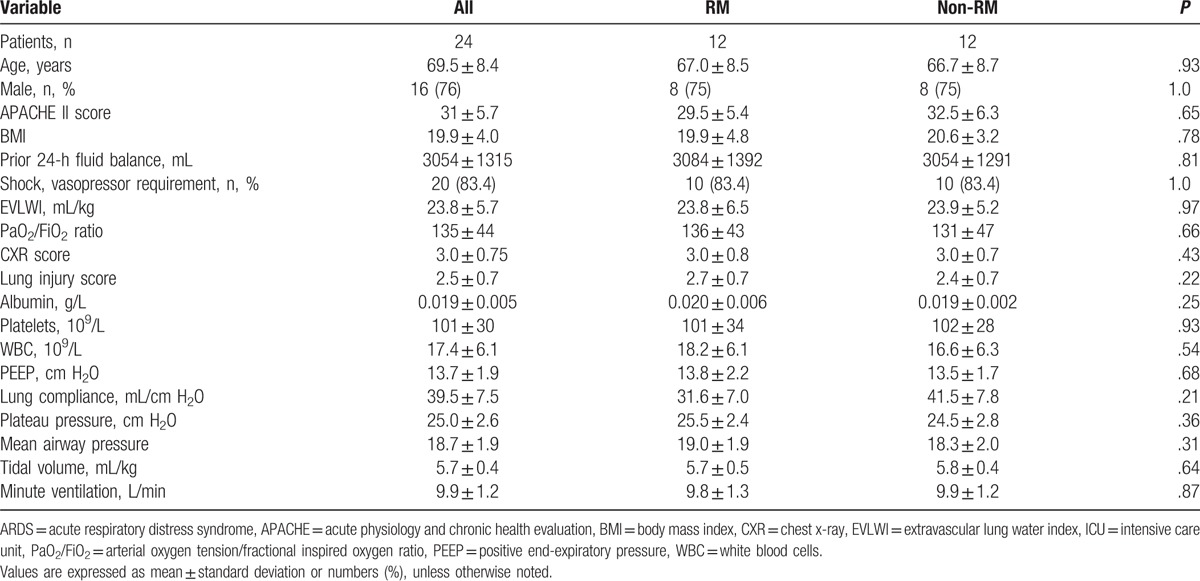
Baseline characteristics of all medical ICU patients with ARDS on day 1.

The study outcomes are presented in Table 2. Ventilator days (20.4 ± 3.8 vs 27.7 ± 5.1 days, *P* = .0006) and ICU stay (22.7 ± 3.8 vs 30.0 ± 5.9 days, *P* = .001) differed significantly between the RM and non-RM groups. ICU mortality (33.3% vs 50%, *P* = .41) and in-hospital mortality (58.3% vs 66.7%, *P* = .67) rates were not significantly different between the RM and non-RM groups.

**Table 2:**
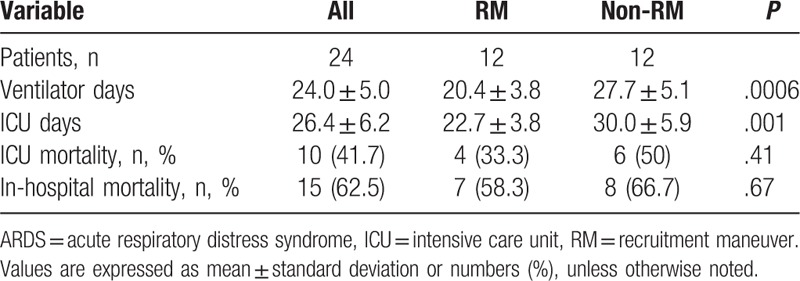
Outcomes of ADRS patients with and without RM.

**Figure 2:**
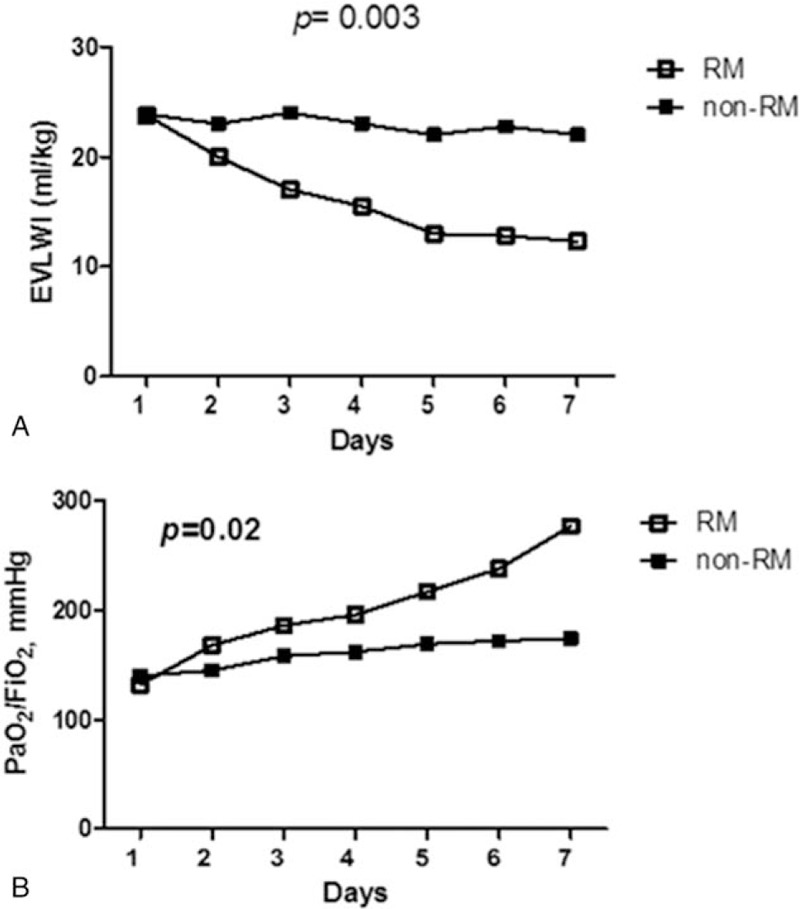
(A) Changes in EVLWI from day 1 to day 7 between the RM and non-RM groups. EVLWI decreased in the RM group, but not in the non-RM group. This was significantly different (*P* = .003, 2-way ANOVA, data presented as means with standard error). (B) Changes in PaO_2_/FiO_2_ from day 1 to day 7 between the RM and non-RM groups. PaO_2_/FiO_2_ improved in the RM group, but not in the non-RM group. This was significantly different (*P* = .02, 2-way ANOVA, data presented as means ± standard error). ANOVA = analysis of variance, EVLWI = extravascular lung water index, PaO_2_/FiO_2_ = arterial oxygen tension/fractional inspired oxygen ratio, PBW = predicted body weight, PEEP = positive end-expiratory pressure, PICCO = pulse contour continuous cardiac output, RM = recruitment maneuver.

Figure 2A shows the changes in EVLWI from day 1 to day 7 between the RM and non-RM groups. EVLWI decreased in the RM group, but remained unchanged in the non-RM group; this difference was statistically significant (*P* = .003 by 2-way ANOVA).

Figure 2B shows the changes in PaO_2_/FiO_2_ from day 1 to day 7 between the RM and non-RM groups. PaO_2_/FiO_2_ improved in the RM group, but did not change in the non-RM group; this difference was statistically significant (*P* = .02 by 2-way ANOVA).

**Figure 3:**
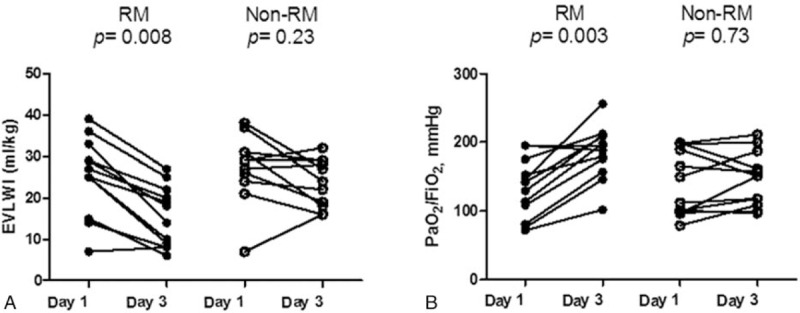
(A) Changes in EVLWI between day 1 and day 3 in the RM and non-RM groups. In the RM group, the EVLWI on day 3 was improved over that of day 1 (25.3 ± 9.3 vs 15.5 ± 7.3 mL/kg, *P* = .008, by the *t* test). In the non-RM group, EVLWI on day 3 remained unchanged when compared to that of day 1 (27.0 ± 5.5 vs 23.5 ± 5.5 mL/kg, *P* = .23, by the *t* test). (B) Changes in PaO_2_/FiO_2_ between day 1 and day 3 in the RM and non-RM groups. In the RM group, the PaO_2_/FiO_2_ on day 3 was improved over that of day 1 (132.3 ± 43.5 vs 185.6 ± 38.8 mL/kg, *P* = .003, by the *t* test). In the non-RM group, the EVLWI on day 3 did not change relative to that of day 1 (140.5 ± 47.1 vs 146.7 ± 39.1 mL/kg, *P* = .73, by the *t* test). EVLWI = extravascular lung water index, PaO_2_/FiO_2_ = arterial oxygen tension/fractional inspired oxygen ratio, RM = recruitment maneuver.

Figure 3A shows a difference in EVLWI between day 1 and 3 in the RM but no difference in non-RM groups. In the RM group, the EVLWI on day 3 was improved over that of day 1 (25.3 ± 9.3 vs 15.5 ± 7.3 mL/kg, *P* = .008 by the *t* test). In the non-RM group, the EVLWI on day 3 did not change relative to that on day 1 (27.0 ± 5.5 vs 23.5 ± 5.5 mL/kg, *P* = .23 by the *t* test). Figure 3B shows the difference in PaO_2_/FiO_2_ between day 1 and 3 in the RM; however, there was no difference in the non-RM group. In the RM group, the PaO_2_/FiO_2_ on day 3 was improved over that on day 1 (132.3 ± 43.5 vs 185.6 ± 38.8 mL/kg, *P* = .003 by the *t* test). In the non-RM group, the PaO_2_/FiO_2_ on day 3 remained unchanged compared to that on day 1 (140.5 ± 47.1 vs 146.7 ± 39.1 mL/kg, *P* = .73 by the *t* test).

## 4 Discussion

This study demonstrated that the alveolar RM improves the EVLWI and oxygenation (PaO_2_/FiO_2_) in patients with ARDS. EVLWI is associated with a reduced capability of RM to improve arterial oxygenation, therefore making it necessary to explore alternative interventions to stabilize hypoxemia during ARDS. PICCO is a suitable technology that provides a quantitative indicator of EVLW at the bedside.^[18,19]^ In this study, we used PICCO to observe the effects of RM on respiratory mechanics and EVLWI in patients with ARDS. In ARDS, the primary mechanisms of lung atelectasis increase the interstitial hydrostatic pressure and pulmonary weight, resulting in worsened lung compliance.^[21–23]^ The use of spiral computed tomography has revealed that RM can improve injured areas adjacent to a foci of consolidated lung tissue.^[23]^

Disturbance of the ventilation-perfusion ratio caused by redundant EVLW contributes to serious hypoxemia and a high fatality rate associated with ARDS. Low-tidal-volume ventilation can reduce ARDS mortality.^[24,25]^ Alveolar RM is 1 method used for the treatment of collapsed and consolidated lungs in patients with ARDS with different etiologies. A variety of RM practices has been reported with different adverse effects and benefits ^[26–28]^. The most important principle of RM is to increase airway pressure in the consolidated lung parenchyma and reopen the atelectatic lung.^[11]^ A short-term sustained inflation pressure of up to 40 cm H2O for 40 seconds is a simple and well-studied RM method commonly used in patients with ARDS.

Our study showed that RM resulted in a substantial improvement in PaO_2_/FiO_2_ and EVLWI in Patients with ARDS, thus decreasing the length of ICU stay and days of ventilator use, despite the lack of significant improvement in ICU and in-hospital mortality rates. This is consistent with other studies.^[7,29]^ However, it is encouraging that the EVLWI and oxygenation (PaO_2_/FiO_2_) improved after RM. The lack of a significant difference in the non-RM group can be explained by the efficacy of RM in reducing EVLWI and improving the PaO_2_/FiO_2_ in ARDS. The PaO_2_/FiO_2_ may decrease to baseline as quickly as 30 to 45 minutes after adjusting the PEEP.^[30,31]^ Nevertheless, in the RM group, the improvement in the average PaO_2_/FiO_2_ occurred over several days, with the greatest improvement during the first 3 days after RM. However, this was not observed in the non-RM group (Fig. 3B). The stability of the alveolar re-expansion may also be limited by the technique used to detect the optimal PEEP. The adjustment of an optimal PEEP using the pressure-volume (P-V) curve, as used in this study, is one of the most widespread and preferable methods used at the bedside.^[32]^ However, particularly in patients with “stiff” lungs due to severe ARDS, the lower inflection point of the P-V curve may be difficult to discern.^[33]^

In ARDS, damage to the pulmonary microvasculature primarily increases the permeability of the endothelial membrane to fluids, resulting in capillary leakage and accumulation of EVLW. The increased EVLWI, in turn, may result in arterial hypoxemia. Our study showed that EVLWI and PaO_2_/FiO_2_ was lower on day 3, when compared to that of those who did not undergo RM. In addition, decreased EVLWI after RM indicates an improvement of pulmonary permeability with improved oxygenation, which was also observed in the RM group. It is possible that RM increased lung water clearance to improve the distribution of all gases in the lung and oxygenation. RM may also reverse the decrease of pulmonary surfactant, reduce alveolar epithelial and endothelial cell injury, and further improve pulmonary vascular function.^[34,35]^ RM may also increase the mRNA expression of surfactant protein in the lung.^[36]^

Our study has several limitations; most notable is the small sample size. A small number of patients were enrolled due to difficulties with enrollment, which included having only 4 to 6 patients in the ICU that were being cared for by the attending physician, not meeting inclusion criteria, and family refusal of enrollment for the RM. Therefore, further large-scale prospective studies are warranted to confirm the relation between EVLWI and alveolar recruitment observed in the present study. Second, the definition of ARDS changed during the study. The investigators could not modify their criteria once the study started, but it was commented on this in the discussion with regard to any effects on interpreting the trial into current practice. Third, Michard reported that the limitations of the dilution methods may lead to an underestimation of EVLW in a large pulmonary vascular obstruction, focal lung injury, and lung resection; however, dilution methods remain the easy and clinically acceptable estimations of EVLW in most critically ill patients, including those with ARDS.^[37]^

RM can be accomplished through various methods.^[11,38]^ Therefore, the results of this study may be inconsistent with other studies using different RM methods. However, findings of the study revealed that RM could improve EVLW, oxygenation, and compliance. Other reports also indicate that a compliance value of >30 mL/cm H_2_O may show a better PF ratio response to RM.^[11,14]^

In conclusion, the alveolar RM is a feasible method for improving oxygenation and EVLWI in patients with ARDS, as well as decreasing ventilator use and the length of ICU stay. Furthermore, the results of this study may aid in developing biomarkers and treatment strategies for of ARDS. EVLWI-lowering therapy with RM may improve oxygenation and vascular permeability in ARDS. Future research is needed to determine the possible underlying mechanisms of ARDS and vascular permeability and develop a novel treatment. In addition, further large-scaled investigations are required to confirm these findings and determine the utility of RM as a tool to improve oxygenation and EVLWI in patients with ARDS.
